# INTERCEPT‐AD: A Gender Analysis of the Phase 1 Experience Among Trial Participants with Early Alzheimer’s Disease and Their Study Partners

**DOI:** 10.1002/alz.089606

**Published:** 2025-01-09

**Authors:** Kelly Johnston, Carrie Presnall, Elizabeth Merikle, Stephanie Cline, Robyn Moxon, Todd Feaster, Eric Siemers

**Affiliations:** ^1^ Fortrea, Inc., Durham, NC USA; ^2^ Acumen Pharmaceuticals, Charlottesville, VA USA

## Abstract

**Background:**

Qualitative patient interviews are increasingly conducted alongside clinical trials; interviews administered early in drug development can yield insight into the patient journey. Examination among patient subgroups may identify factors that influence the patient experience, including trial participation. As part of the INTERCEPT‐AD phase 1 study evaluating the safety and tolerability of the Aβ oligomer‐targeting monoclonal antibody ACU193, we conducted semi‐structured qualitative interviews among a subset of participants with mild cognitive impairment (MCI) or mild dementia due to Alzheimer’s disease (AD) and their study partners to obtain feedback on their trial experience and the decision‐making process preceding trial enrollment; results were compared between study participant genders.

**Method:**

A subset of study participants and study partners completed qualitative interviews following the final study visit. Topics included referral source, motivations for participating, enrollment decision‐making, concerns regarding study medication and procedures, and positive and negative aspects of participation. Study participant/study partner dyads were defined as female or male according to the gender of the study participant as it was captured in the main study protocol; study partner gender was not obtained. Transcripts were coded and analyzed using principles of qualitative thematic analysis and grounded theory. Differences in concepts mentioned between genders were reported when >20%, and when sample size was sufficient (≥5 dyads reporting a concept with a difference of >2 mentions between genders).

**Result:**

Twenty‐eight participants (64.2% female) and their study partners were interviewed, representing 43% of the total randomized trial population (n = 65; 53.8% female). Female dyads accounted for 62% and 70% of those who first heard about INTERCEPT‐AD from social media or physicians, respectively. More female dyads desired additional information about AD (86% of all dyads reporting this concept), to benefit others (75%), and to benefit self (65%) by participating. Regarding the decision to enroll, those who decided independently were exclusively female dyads; female dyads accounted for 71% among those who had pre‐trial concerns about side effects. More female dyads expressed dislike for cognitive tests (83%) and blood draws (78%).

**Conclusion:**

Certain factors of trial participation differed by participant gender, including motivations for participation and perceived burden of some study procedures.

